# BECOMING A DEMENTIA CARE PARTNER DURING THE COVID-19 PANDEMIC

**DOI:** 10.1093/geroni/igac059.266

**Published:** 2022-12-20

**Authors:** Hanzhang Xu, Laura Fish, Raman Nohria, Jacob Christy, Margaret Falkovic, Matthew Dupre, Ruth Anderson, Truls Østbye

**Affiliations:** Duke University, Durham, North Carolina, United States; Duke University School of Medicine, Durham, North Carolina, United States; Duke University School of Medicine, Durham, North Carolina, United States; Duke University School of Medicine, Durham, North Carolina, United States; Duke University School of Medicine, Durham, North Carolina, United States; Duke University, Durham, North Carolina, United States; UNC Chapel Hill, Chapel Hill, North Carolina, United States; Duke University School of Medicine, Durham, North Carolina, United States

## Abstract

Persons with newly diagnosed Alzheimer’s disease and related dementias (ADRD) and their care partners confront multiple challenges. These challenges have been even greater during the COVID-19 pandemic, where supportive resources often are limited or even discontinued. We conducted semi-structured interviews with 21 care partners of persons who were recently diagnosed with ADRD (2019-2020) to explore their lived experiences of adjusting to the new role. Directed and conventional content analyses were used and were informed by the life course theory. Care partners perceived difficulty in accessing medical and social services for their loved ones, particularly during the pandemic. Despite experiencing distress, some care partners chose not to seek help for fear of contracting COVID-19. This study provides insights on the unmet needs of care partners during a pandemic and highlights that effective, long-term strategies are needed to continue providing person-centered care to persons with ADRD and their families.

